# A systematic comparison of generative models for medical images

**DOI:** 10.1007/s11548-022-02567-6

**Published:** 2022-02-07

**Authors:** Hristina Uzunova, Matthias Wilms, Nils D. Forkert, Heinz Handels, Jan Ehrhardt

**Affiliations:** 1 Artificial Intelligence in Medical Imaging, Lübeck, Germany; 2 Institute of Medical Informatics, University of Lübeck, Lübeck, Germany; 3 Department of Radiology, University of Calgary, Calgary, Canada

**Keywords:** Comparison, Generative models, Shape and appearance models

## Abstract

**Purpose:**

This work aims for a systematic comparison of popular shape and appearance models. Here, two statistical and four deep-learning-based shape and appearance models are compared and evaluated in terms of their expressiveness described by their generalization ability and specificity as well as further properties like input data format, interpretability and latent space distribution and dimension.

**Methods:**

Classical shape models and their locality-based extension are considered next to autoencoders, variational autoencoders, diffeomorphic autoencoders and generative adversarial networks. The approaches are evaluated in terms of generalization ability, specificity and likeness depending on the amount of training data. Furthermore, various latent space metrics are presented in order to capture further major characteristics of the models.

**Results:**

The experimental setup showed that locality statistical shape models yield best results in terms of generalization ability for 2D and 3D shape modeling. However, the deep learning approaches show strongly improved specificity. In the case of simultaneous shape and appearance modeling, the neural networks are able to generate more realistic and diverse appearances. A major drawback of the deep-learning models is, however, their impaired interpretability and ambiguity of the latent space.

**Conclusions:**

It can be concluded that for applications not requiring particularly good specificity, shape modeling can be reliably established with locality-based statistical shape models, especially when it comes to 3D shapes. However, deep learning approaches are more worthwhile in terms of appearance modeling.

## Introduction

Building representative, generative models that capture shape and appearance variations of anatomical structures commonly observed in a population of subjects, is a classical problem in computational anatomy. Such models have multiple applications in medical image analysis where they can, for example, provide prior information about plausible shape and appearance configurations in classical image segmentation and registration approaches [13, 17]. In an era where medical image analysis is dominated by deep learning-based methods, models of shape and appearance are still valuable as either a way to systematically generate additional training data [15, 29] or to directly integrate such information into the network architecture [20, 33].

Statistical shape models (SSMs) use the principal component analysis (PCA) on point-wise shape representations to compactly describe the shape variability [4, 12].

While those models have had great success in the past [12], they come with significant disadvantages as they are only able to represent linear manifolds, rely on point-by-point correspondences across all training shapes, and do not generalize well to unseen data when only few training samples were available. Some of those shortcomings have been addressed via targeted extensions of the core method. For example, in [17] a nonlinear extension of SSMs is derived, [5, 32] introduce methods to still learn representative SSMs based on few real samples, and [14, 18] propose a probabilistic method to build SSMs without fixed point-by-point correspondence. In addition to shape modeling, the PCA-based mechanisms underlying SSMs [4] have also been used to learn statistical appearance models (SAMs) that simultaneously model shape and grayvalue appearance of anatomical structures [3, 18]. This not only allows for an improved representation of the structures being modeled, but also enables the generation of realistic images for tasks like data augmentation when training neural networks [29].

In recent years, the focus has shifted away from traditional SSMs or SAMs for generative modeling in medical image analysis as deep learning approaches have become more popular. For example, autoencoders (AEs) and variational autoencoders (VAEs) were successfully applied for feature extraction [2] or unsupervised pathology localization [28]. Furthermore, so-called generative adversarial networks (GANs) known for their excellent ability to generate realistic images have been utilized for data augmentation [7]. Moreover, the recently developed diffeomorphic and deformable autoencoders (DAEs) share foundations with SSMs and are thus particularly suitable for representation learning [1, 26]. When compared to traditional SSMs and SAMs, deep learning-based generative models have many advantages in terms of model flexibility and image generation. For example, typically no separate handling of shape and appearance is required, point-by-point correspondences are not needed, and they are flexible enough to directly handle nonlinear relationships. However, deep learning-based models typically require a much larger amount of training data than their traditional counterparts and are often deemed as black boxes that are hard to interpret.

Given the amount of various traditional and deep learning-based shape modeling methods, a systematic investigation of their specific strengths and drawbacks in relevant medical image analysis scenarios has been missing so far. This work aims to consistently and systematically evaluate the performance and characteristics of two traditional approaches (SSMs and their locality-based extension [32] (LSSMs)) and four deep learning-based autoencoding architectures on public datasets. Although the models differ with respect to the representation of input and output data, they were chosen since they agree in terms of purpose and aim: generative modeling of shape and/or appearance information based on a set of training data. Despite the fact that shape information is encoded as contours (or pseudo-landmarks) in statistical shape models, and as label images in CNN-based approaches, all of the discussed approaches can be used interchangeably in downstream tasks, e.g., to synthesize training data, to provide shape priors, or for data augmentation [6, 7, 29]. The provided comparison aims to contribute to the discussion which generative model is the most appropriate for a given task by evaluating their reconstruction performance as well as the specificity and diversity of generated samples depending on the size of the training dataset.

Furthermore, we study and compare the consistency and characteristics of the respective latent spaces. In a previous study [27], we examined the shape modeling properties of SSMs and different neural networks for 2D shapes. We were able to show that on average neural networks perform on par with SSM in terms of generalization ability while outperforming them in terms of specificity. On the other hand, traditional SSMs and specific extensions especially showed their benefits in scenarios with small training populations. This work substantially extends [27] by (1) also investigating 3D shapes in addition to 2D data, by (2) incorporating appearance information, and by (3) utilizing additional metrics to inspect sample quality and latent space characteristics. We strongly believe that those extensions of our analysis will help to obtain a better understanding of the differences between various deep learning and classical shape and appearance modeling approaches.

## Methods

### Compared approaches

In this work, the following two statistical and four deep-learning approaches are considered in a systematic comparison.

*Statistical Shape and Appearance Models* Statistical shape models (*SSMs*) are built using a training set of *N* discrete, vectorized shape representations $\mathbf {x}_1 \dots \mathbf {x}_N$ [4].

Here, each $\mathbf {x}_i\in \mathbb {R}^{dm}$ is composed of *m* landmarks representing the object’s shape in a *d*-dimensional space. PCA of a training set is used to create a mean shape $\mathbf {x}_\mu$ and an orthonormal basis $\mathbf {U}\in \mathbb {R}^{dm\times p}$ for projecting shape representations into a low-dimensional latent space $\mathbf {z}\in \mathbb {R}^p$ via $\mathbf {x}_{new}=\mathbf {x}_\mu + \mathbf {U}\mathbf {z}$ or to generate new shapes by varying $\mathbf {z}$.

SSMs can be expanded by appearance modeling (*SAMs*) [3] by considering appearance images $\mathbf {a}_i$ and warping their shape representations $\mathbf {x}_i$ to $\mathbf {x}_\mu$ with the corresponding transformation $\varphi _i$. The resulting ”shape-normalized” images can be used in a similar manner for a PCA-based modeling of the intensities sampled on multiple points. After the sampling of the appearance parameters, the resulting image need to be warped back with $\varphi ^{-1}$.

*Locality-based Statistical Shape and Appearance Models* In classical SSMs, the number of training samples influences the flexibility of the model, since the size of the latent space is limited by the size of the training set. Locality-based SSMs (*LSSMs*) [32] introduce additional flexibility by breaking global relationships and assuming that local shape variations have limited effects in distant areas. This idea can be integrated into the traditional SS(A)M framework by manipulating covariances based on the distance between landmarks in a multi-resolution manner.

*Autoencoders* Autoencoders (*AEs*) are typically neural networks that aim to learn a low-dimensional representation from high-dimensional input data [16]. They consist of an encoder $f : \mathbb {R}^d\rightarrow \mathcal {Z}$ that maps the input $\mathbf {x}\in \mathbb {R}^d$ to a latent space variable $\mathbf {z}\in \mathcal {Z}$; and a decoder $g: \mathcal {Z} \rightarrow \mathbb {R}^d$ that aims to reconstruct the input image as flawlessly as possible by only considering its latent representation. To ensure a good reconstruction of the input image, a reconstruction loss between the real input image and its reconstruction is usually calculated.

AEs can be observed as generative models, where an unseen sample $\mathbf {x}_{new}$ can be reconstructed as $\mathbf {x}_{new}\approx g(f(\mathbf {x}_{new}))$ and new samples can be generated by sampling a random latent vector $\mathbf {z}_{sample}$ and computing $g(\mathbf {z}_{sample})$. However, using AEs for the generation of new samples in this manner might be infeasible due to the unknown underlying distribution of the latent space. Thus a random sample can lie far away from the learned distribution and produce unrealistic results.

*Variational Autoencoders* Variational autoencoders (*VAEs*) [16] are an extension of conventional AEs that prevent the problem of unknown latent distribution. VAEs constrain the latent space to a known prior distribution $p(\mathbf {z})$, most commonly set to the normal distribution $\mathcal {N}(0,1)$. In practice, this is done by applying a Kullback-Leibler (KL) loss to the latent space. Using VAEs for the reconstruction of unseen samples can be done analogously to conventional AE; however, the main difference lies in the generation of new samples by sampling a $\mathbf {z}_{sample}\sim \mathcal {N}(0,1)$.

*Autoencoder Generative Adversarial Nets* Autoencoder generative adversarial nets (*AE-GANs*) [19] are a combination of an autoencoder with an adversarial discriminator inspired by GANs [9]. Thus an AE-GAN consists of an encoder $f: \mathbb {R}^d\rightarrow \mathcal {Z}$, a decoder $g: \mathcal {Z} \rightarrow \mathbb {R}^d$ and enclosed adversarial discriminator $d:{\mathbb {R}}^d\rightarrow${**real**,**fake**}. Here, the typical GAN-loss is combined with a reconstruction loss to improve convergence. For our purposes, the straight-forward use of GANs is infeasible, since they do not directly enable reconstruction of samples and approaches like [24] are time-consuming. Using an AE-GAN architecture enables the reconstruction of samples analogously to AEs.

*Diffeomorphic Autoencoders* Similarly to statistical models, diffeomorphic autoencoders (*DAEs*) [1] assume images as a deformed version of a global template. DAEs also feature an encoder-decoder structure; however, the decoder’s output is a deformation field $\varphi$, such that given a template $\mathbf {t}$ the input image $\mathbf {x}$ can be reconstructed as $\mathbf {t}\circ \varphi$, where $\circ$ denotes the warping function. Diffeomorphic autoencoders most commonly constrain the latent space to a normal distribution using a KL loss.

Using the annotations from Sect. 2.1, unseen samples can be reconstructed as $\mathbf {x}_{new}\approx \mathbf {t}\circ g(f(\mathbf {x}_{new}))$ and new samples can be generated as $\mathbf {t}\circ g(f(\mathbf {z}_{sample}))$ with $\mathbf {z}_{sample}\sim \mathcal {N}(0,1)$. DAEs reliably capture shape changes between samples; however, modeling of appearance is not feasible with this approach. For the additional modeling of intensity offsets, an extension to the DAE approach similar to [26] is used in this work. We correspond to this model as appearance DAE (*ADAE*). The main idea is to represent images as $\mathbf {x}\approx (\mathbf {t}+\mathbf {a})\circ \varphi$ with $\mathbf {a}$ being an image-specific pixel-wise intensity offset. To enable this, the encoder *f* generates a composite latent representation $\mathbf {z}=[\mathbf {z}_s, \mathbf {z}_a]$ for shape and appearance respectively and two decoders $g_s$ and $g_a$ each generate a shape displacement $\varphi$ and an appearance map $\mathbf {a}$.

*Architectures and Implementation Details*

In order to decouple architectural search from network performance and enable comparability between the deep learning approaches in terms of the number of trainable parameters, the same architectures (except for particular model-specific components) were applied for all models. In this way, the expressiveness of the networks for a fixed architecture can be evaluated and separated from the performance gain due to architectural optimization. Each encoder (or discriminator) contains three convolutional layers (2D or 3D depending on the input data) with stride two and a growing number of channels [${in_{ch}}$,20,40,80] and a fully connected layer at the end to obtain the latent vector. The decoders contain three subsequent bilinear upsampling and convolutional layers with a decreasing number of channels [80,40,20,${out_{ch}}$]. The input and output number of channels ${in_{ch}}$ and ${out_{ch}}$ depends on the input and output types (one-hot encoded label images, displacement fields or one-channel intensity images). When modeling shapes (represented as labels) a weighted generalized Dice loss is used [25], when modeling appearances an SSIM-loss is chosen. For the diffeomorphic autoencoders, the displacement field is regularized using diffusion and L1-regularization as in [26]. All parameters for the weighting of the loss function were taken from [26], where both regularization terms are weighted by a factor of ten.

### Evaluation techniques

#### Assessing the quality of generative models

*Generalization Ability* Generalization ability (GA) is the ability of a generative model to reconstruct samples unseen during training. Formally, given a set of real left out images $\mathbf {r}_i\in \mathcal {R}$, GA is measured as $\frac{1}{N_R}\sum _i^{N_R} dist(\mathbf {r}_i, \varvec{\hat{r}}_{i})$, where $dist(\cdot ,\cdot )$ is a suitable image-wise distance metric and $\varvec{\hat{r}}$ is the reconstructed input $\mathbf {r}$. The reconstruction of unseen samples is established depending on the used approach as explained in 2.1.

*Specificity* The specificity is the ability of a generative model to generate new samples that are similar to the real samples of the training dataset, i.e., realistic samples can be generated. Specificity is measured by generating a dataset $\mathbf {s}_j\in \mathcal {S}$ of $N_S$ sampled images and calculating: $\frac{1}{N_S}\sum _j^{N_S} \min _{\mathbf {r}_i\in \mathcal {R}} \{dist(\mathbf {r}_i, \mathbf {s}_{j} | \mathbf {r}_i\in \mathcal {R}\}$ where $\mathcal {R}$ is a set of real images. Specificity measures the distance of a generated image to its best fitting real image; however, the diversity of the generated samples is not considered. This means that a good specificity may indicate that the model only generates one image that suits a particular real image very well. Thus the diversity of the generated images is also considered by the following evaluation method.

*Likeness* The likeness score proposed by [10] evaluates the realism of generated images by considering the aspects creativity, inheritance and diversity. The authors propose to compute a distance-based separability index (DSI) by comparing the distributions of distances between images of the same class (real or synthetic) and different classes. For two sets $\mathcal {R}$, $\mathcal {S}$ of real and synthetic images, the intra-class distance set (ICD) is defined as: $\{d_\mathcal {R}\}=\{dist(\mathbf {r}_i, \mathbf {r}_j)| \mathbf {r}_i, \mathbf {r}_j \in \mathcal {R}; \mathbf {r}_i \ne \mathbf {r}_j\}$ (analogously $\{d_\mathcal {S}\}$ for $\mathcal {S}$), and the between-class distance set (BCD) can be determined as: $\{d_{\mathcal {R},\mathcal {S}}\}=\{dist(\mathbf {r}_i, \mathbf {s}_j)| \mathbf {r}_i \in \mathcal {R}; \mathbf {s}_j \in \mathcal {S}\}$. The Kolmogorov–Smirnov statistic is used to quantify the similarity of the distributions $s_\mathcal {R}=KS(\{d_\mathcal {R}\},\{d_{\mathcal {R},\mathcal {S}}\}), s_\mathcal {S}=KS(\{d_\mathcal {S}\},\{d_{\mathcal {R},\mathcal {S}}\})$, and the average of the two KS similarities defines the DSI: $DSI(\mathcal {R},\mathcal {S})=(s_\mathcal {R}+s_\mathcal {S})/2$. We refer to this metric as *likeness* since it describes the similarity between the distribution of the real data and the generated data. The likeness ranges from 0 to 1 where smaller values indicate better synthetic images.

**Fig. 1:**
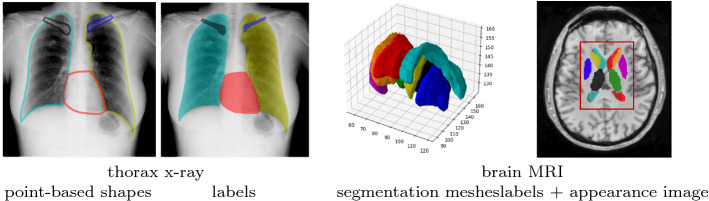
Example image data used in our experiments

#### Quantifying the latent space

*Compactness* The most important property of the latent space is its dimension, with a smaller number of latent dimensions contributing to the interpretability of the model. While in SSMs the dimension of the latent space is determined by the number of modes describing the desired percentage of variability (95%), for deep learning models this must be empirically determined a priori (see appendix for choice of latent dimensions).

*Normality* An important assumption of all approaches is that new samples can be generated by sampling a vector $\mathbf {z}$ from a normal distribution. This requires a roughly normal distribution of the learned latent spaces. To assess this property, we encode a set of real samples and examine the distributions along each dimension using a Shapiro-Wilk test [23]. In this manner, the percentage of not-normally distributed components of the latent space can be determined. A further visual evaluation of the smooth distribution of the latent space can be established by interpolating between the projections of two random images and visualizing the decodings of the intermediate latent vectors.

*Latent ambiguity score* A desired property of generative models is that if an image $\mathbf {x}$ is mapped to its latent variable $\mathbf {z}$ and reconstructed to $\varvec{\hat{x}}$, the reconstruction $\varvec{\hat{x}}$ should also get mapped to the same latent vector. This is given for SSM-based models, however not mandatorily fulfilled by deep learning approaches. To asses the extent of the problem, we propose the latent ambiguity score $LAS=\mathcal {\overline{D}}/\mathcal {\overline{D}}_{base}$, where $\mathcal {\overline{D}}=\frac{1}{N_R}\sum _i^{N_R} ||f(\mathbf {r}_i)-f(g(f(\mathbf {r}_i)))||_2$ is the mean latent space distance of images $\mathbf {r}_i \in \mathcal {R}$ and their reconstructions, and $\mathcal {\overline{D}}_{base}=\frac{1}{N_R \cdot (N_R-1)}\sum _i^{N_R}\sum _{j\ne i}^{N_R}||f(\mathbf {r}_i)-f(\mathbf {r}_j)||_2$ is the mean encoded distance between different images. LAS close to zero corresponds to an unambiguous latent space, whereas a score close to one indicates that the latent mappings of the real and the reconstructed images are nearly randomly located. Thus models with high LAS are able to sample the entire latent space by simply subsequently inputting the reconstructions of the previous input.

## Experiments and results

### Data and experimental setup

*Thorax X-Rays* We use an openly available thorax X-ray dataset containing 247 chest radiographs and ground truth segmentations of five structures: the heart, both lungs and both clavicles (see Fig. 1 for examples) [30]. This dataset is only used for shape modeling, thus for the SSM methods, point-based representations are used, and for the deep-learning approaches the segmentation labels are applied directly. The images are split in 123/114/10 test/train/validation.

*Brain MRIs* This dataset contains 600 3D T1-weighted brain MRIs from the publicly available IXI dataset^1^ [11]. Anatomical labels were generated using atlas-based segmentation, while simultaneously obtaining the one-to-one correspondences needed for SSM-based methods from the registration. A dataset split of 300/290/10 test/train/validation is used. For this dataset, we explore both shape and appearance modeling of the given segmentations and intensity volumes. Since deep-learning methods are very resource demanding, the images and labels are cut to a central area around the ventricles of size $64 \times 96 \times 64$ resulting in eight labels as shown in Fig. 1.

Three experiments are carried out: 1) shape modeling for 2D thorax x-rays; 2) shape modeling for 3D brain MRIs; 3) appearance modeling for 3D brain MRIs. For the shape modeling scenarios, point-based representations of the segmentation labels are used as input, whereas the deep-learning methods take in the one-hot-encoded labels directly. For the appearance modeling scenario, the intensity image volumes can be directly used by the neural networks; however, the SAMs consider shape distortions (given by registration displacement fields) and appearances separately. Each model is trained on various training set sizes and the generalization ability, specificity and likeness are assessed in accordance to that. This allows an observation of the quality of the generative models depending on the training set size and thus an assessment of their robustness. To avoid choosing unsuitable training data for smaller training set sizes, a multi-fold training is performed (four folds for $N<100$, two folds for $N\ge 100$), averaging the results over the folds. To establish fairness against all methods when generating new samples, the latent vectors are drawn from the normal distribution described by the mean and standard deviation of the latent vectors of the training dataset. The metrics used for assessing the generative models vary depending on the data. When shapes are considered, average symmetric surface distance (ASSD) is calculated for all models. Here, a geometric distance is deliberately chosen since a landmark distance is infeasible for the deep learning approaches using labels as inputs, and a label-based metric such as Dice is not precise enough and might favor the methods trained on labels since they optimize towards a generalized Dice coefficient during training. Hence, the chosen ASSD is the middle ground between the slight loss of accuracy by computing the contours from the landmarks for the evaluation of statistical methods and the conversion from contours to labels and backward for the deep learning approaches. For the appearances, the L1 distance is used here. Since our experience showed that metrics like MSE and SSIM behave similarly, those results are only shown in Appendix. The latent space evaluation is carried out for the largest possible training sets for each experiment.

**Fig. 2:**
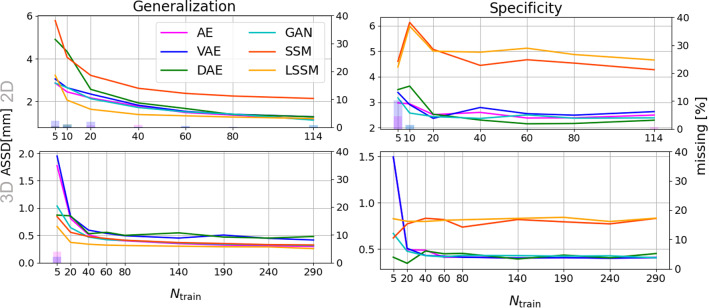
Generalization ability (left) and specificity (right) for the 2D (top) and 3D shapes (bottom) measured in ASSD (left y-axis) – smaller values are better. The bars indicate the percentage of not generated structures (right y-axis)

**Fig. 3:**
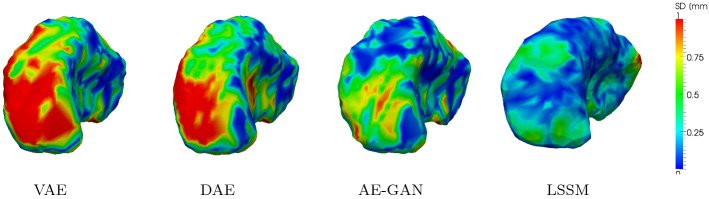
Absolute surface distances from a reconstructed left putamen (blue segmentation label in Fig. 1) to the original input. Smaller values indicate better reconstruction

### Results

*2D and 3D Shape Modeling*

The specificity and generalization ability for the shape models are presented in Fig. 2. For the 2D scenario, all deep-learning approaches perform similarly to the LSSM in terms of generalization ability, whereas the classical SSM performs significantly worse, regardless the training set size. For small training set sizes ($N\le 40$), LSSM even outperforms the deep-learning methods. Furthermore, for small training set size the neural networks tend to not generate small structures like the clavicles (bars in Fig. 2). This problem does not appear when using (L)SSMs or DAEs, since they represent shapes as a deformed global template that contains all structures. When it comes to specificity, the deep-learning methods outperform the (L)SSMs for all training set sizes.

A different behavior can be observed for the 3D shapes. Here, the (L)SSMs both yield slightly improved generalization ability compared to the deep-learning methods. Please note that all methods perform with voxel-level accuracy for $N>5$. Again, the deep-learning methods yield better specificity values; however, the difference between the models is not as vast and the distances for all models lie under one voxel. A significant drawback of the neural networks in this scenario, is however their enormous GPU memory demand, which is why the input shape size needed to be drastically decreased in order to enable training on a 12GB GPU for all models. Figure 3 shows surface distances for a reconstructed structure (putamen) by some of the methods, where the LSSM and AE-GAN approaches deliver the smallest distances, which is consistent with the presented quantitative results.

The assessment of the latent spaces gives further insight. The normality test of latent vectors shows that SSMs have a large amount of non-normally distributed dimensions (LSSM: 40%, SSM: 33%) which might be the reason for their bad specificity. On the contrary, the deep-learning methods yield less non-normally distributed dimensions (VAE: 10%, AE: 18%, DAE: 13%, AE-GAN: 19%), whereas expected methods with explicit KL-normalization have the smallest percentage of non-normally distributed dimensions. In terms of compactness, the SSM methods are much more compact, whereas the latent space dimension of the neural networks is much larger, implicitly making them harder to interpret (Table 2).

Table 1 shows the LAS for the different models. SSMs, by definition, have an $LAS=0$; however, all deep learning methods show scores $>0$. Again, neural networks that apply a KL-loss tend to map reconstructions closer to their initial input shapes. Thus, VAEs and DAEs seem to build a less ambiguous latent space than AEs and (AE-)GANs. A less ambiguous latent space is generally desirable in generative modeling; however, in deep learning methods some ambiguity comes with a trade-off concerning the representation of degrees of freedom. Since statistical models are linear mappings and have an a-priori one-to-one correspondence from the input to its latent representation and back, their representation abilities are limited. Following from this, some ambiguity should be taken into account for the higher expressiveness of deep learning models.

**Table 1:** Latent space ambiguity scores (LAS) for all models and training scenarios. Values close to zero indicate an unambiguous latent space

Data	SS(A)M	LSS(A)M	AE	VAE	AE-GAN	(A)DAE
Shapes 2D	0	0	0.4	0.02	0.5	0.03
Shapes 3D	0	0	0.95	0.14	0.45	0.05
Appearance 3D	0	0	0.27	0.06	0.14	0.07

**Table 2:** Compactness for all models and data. Smaller values are better. For the statistical methods, the compactness is specified in a range depending on the training set size. For the models that observe shape and appearance separately, the values are noted as $dim_{s} +dim_{a}$

Data	SS(A)M	LSS(A)M	AE	VAE	AE-GAN	(A)DAE
Shapes 2D	[3,14]	[4,55]	512	512	512	512
Shapes 3D	[4,51]	[31,100]	1024	1024	1024	1024
Appearance 3D	[4+4,257+189]	[37+41,383+351]	1024	1024	1024	1024+64

**Fig. 4:**
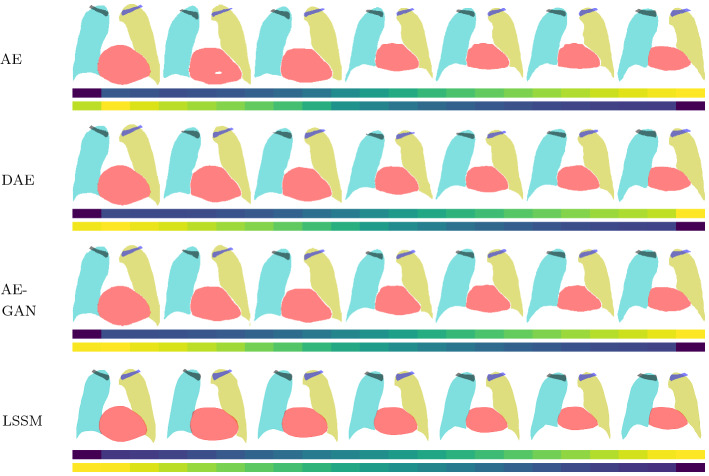
Visualization of the linear interpolation between latent vectors of two shapes (first and last in a row). The bars underneath indicate the ASSD between a reconstruction (20 steps) and the first shape (top bar); and a reconstruction and the last shape (bottom bar). First and last values are the ASSD between real shape to itself or to the second shape and vice versa: yellow(max)$\rightarrow$blue(0). Some models skipped due to space constrains

To further visualize the latent spaces, Fig. 4 shows interpolations between two random 2D shapes. Due to the missing regularization of the latent space, AEs produce holes in the segmentations and they show abrupt, irregular changes between interpolated shapes (middle shape and shape left to it), which are also present to a lesser extent in DAEs and AE-GANs. In contrast, LSSMs show continuous and smooth shape interpolations due to their linear nature.

**Fig. 5:**
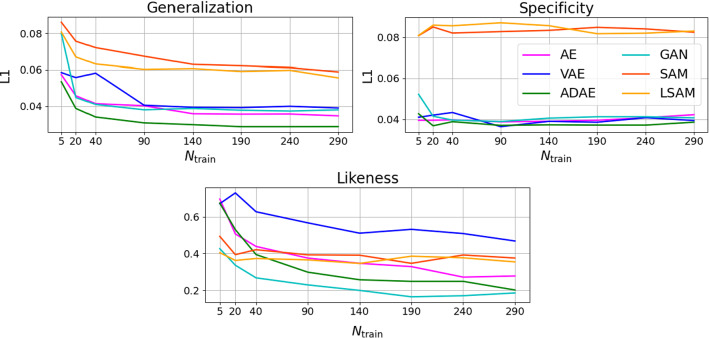
Generalization ability, specificity and likeness for the 3D appearance modeling measured as L1 distances: smaller values are better

*3D Appearance Modeling* Figure 5 shows the results of the 3D appearance modeling. Here, the deep-learning methods perform better in terms of generalization and specificity for all training set sizes, whereas the ADAE yields the best results. In terms of likeness, the VAE yields the worst results, possibly due to the blurriness of the generated samples. The best likeness is delivered by the AE-GAN approach, followed by ADAE that is constrained through a template and thus most likely generates less diverse samples.

**Fig. 6:**
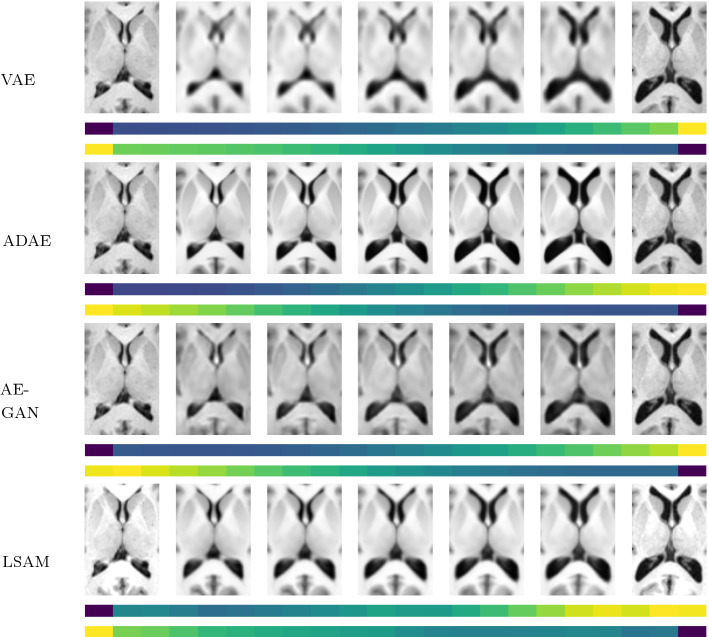
Interpolation experiment for the 3D appearance images (axial slices). Visualization analogous to Fig. 4 (except for measuring L1 distances between images)

In terms of latent space ambiguity and normality, the same tendency as in the previous experiments can be observed. The percentage of non-normality distributed components can be broken down as follows: AE: 12%; VAE: 7%; GAN: 14%; DAE: 7%; PCA: 52%; LPCA: 56%. However, in terms of compactness, the SAM and LSAM show a significant increase of modes, shrinking the gap between the neural networks and the statistical approaches. The interpolation experiment from Fig.  6 also shows that a smooth interpolation is possible for all methods. However, the images generated by the VAE are very blurry. The images generated by the (L)SAMs look more realistic, yet, they are not able to attain the high quality of the images generated by the ADAE and AE-GAN models.

## Discussion and conclusion

In this work, a systematic comparison of the shape and appearance modeling abilities of four deep learning and two statistical generative models was performed. To facilitate comparability, all methods were consistently evaluated using the same image data, processing pipeline and metrics, and the network architectures of the deep-learning methods were kept constant except for model-specific components.

In general, our experiments show that for shape modeling, present extensions of classical SSMs have a number of advantages over deep learning-based approaches. Among the tested methods, the locality-based SSM approach achieves the best generalization ability and requires less training samples and less computational resources, especially in the case of 3D shapes. The latent space of SSM-based models shows advantages like better interpretability and compactness and is producing continuous and smooth interpolation results. Another advantage of SSM-based models in this scenario is that anatomical shapes are represented point-based with sub-voxel accuracy, while CNN-based architectures use voxelized label maps. The main disadvantage of SSM-based models is, however, the lower specificity of generated samples, which is likely caused by the non-normality of the latent space distribution.

Since they use a linear mapping of the input data to the latent vectors, the distribution of the training data defines the distribution of the latent space. Interestingly, even the deep learning methods that use no KL-constrain on their latent space, map the the input data to a latent distribution that has far more normally distributed components than the ones resulting from statistical models. This results into fairly good specificity (e.g. AE-GAN) even though no latent space constrain is used. A possible reason for that is the usage of weight decay regularization during the optimization process which gives the network’s weights a Gaussian prior and thus the latent space is also pushed in the direction of a normal distribution. Here, the regularization term is low weighted; however, other works feature approaches like regularized autoencoders [8] that only use regularization techniques and omit the KL-loss. Yet, the explicit constraining of the latent space to a normal distribution using a KL-term here yields slightly increased specificity; however, this might interfere with the model’s generalization ability and presents an additional loss component, making the optimization process more complex and increasing the number of hyperparameters. Thus, whether a KL-constrain is used greatly depends on the chosen application and desired results.

For the simultaneous shape and appearance modeling, however, the deep-learning methods show considerable advantages: the ADAE and AE-GAN generate diverse, sharp and realistic images and show best results in terms of all metrics. Here, ADAEs have the further advantage that their latent space is more normally distributed and less ambiguous due to the use of the KL divergence. Surprisingly, they also prevent blurriness of the generated samples typical for the standard VAE approach. SAM-based approaches require pre-registration of all training images to establish point-by-point correspondences, and unavoidable registration errors may reduce generalization ability. Also, the compactness and computational resources needed approach those of deep learning methods due to the size of the underlying covariance matrices. So that SAM-based methods are clearly inferior to deep-learning models for appearance modeling.

Given the wide variety of generative models, we only considered a selection of commonly used approaches in this paper. We provided a comparison between LSSMs and other classical shape modeling approaches, like wavelet-based SSMs or Gaussian process morphable models, in our previous work [31, 32]. Other approaches, like autoregressive generative models [21] or normalizing flows [22], show promising generative properties, but are costly to train and have few applications in the medical field so far. Besides the integration of such models in a systematic comparison as focused in this paper, a further interesting line of research is to combine the strengths of different models. One example is included in this paper by combining autoencoders with a GAN-inspired discriminative loss function. Another possible example is to use normalizing flows to ensure normality distributions in the latent space of VAE or SSM models. This will be one of our future directions of research.

In conclusion, this study enables insights into the advantages and disadvantages of generative models for medical images in different settings, providing a decision support for the choice of a suitable model and motivating further research for the improvement of the presented drawbacks. Considering the various possible applications and purposes of generative models, their different characteristics need to be weighed with regard to the specifically planned application, since, e.g., interpretability and a small number of parameters might be an important feature for applications like segmentation but high specificity and diverse and naturalistic samples might be required for data augmentation for the training of neural networks.

## Figures and Tables

**Fig. 7:**
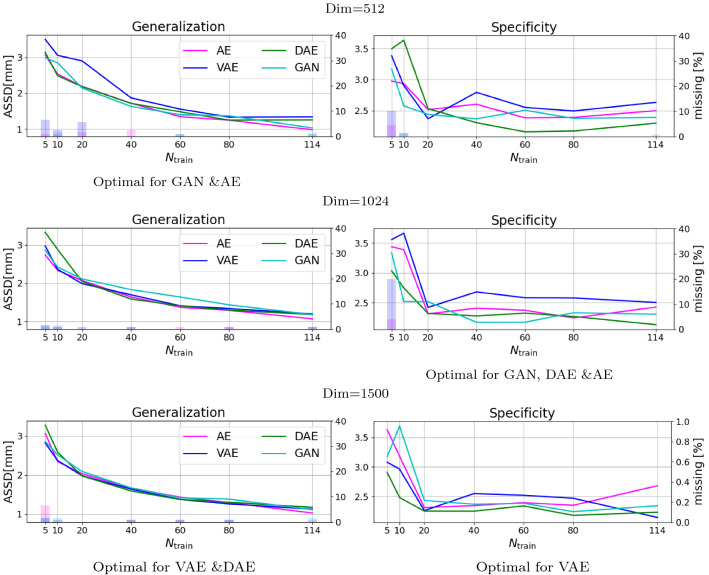
The results for the 2D shapes plotted for different latent dimensions. The dimensions optimal for a certain model and metric are noted underneath the plots, respectively

**Fig. 8:**
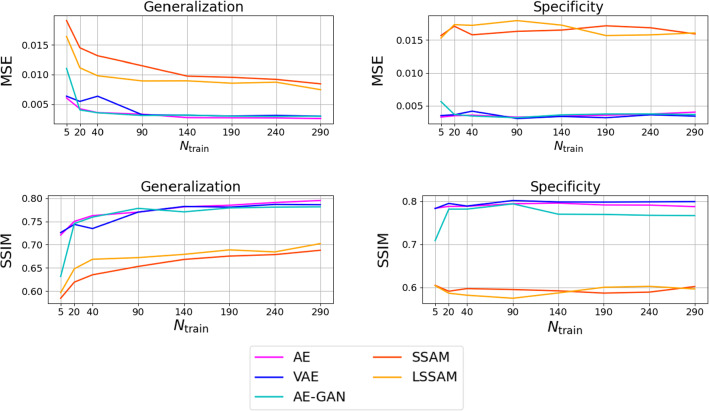
Generalization ability and specificity of all models measured in MSE($\downarrow$) and SSIM($\uparrow$). For colors, see legend (bottom)

## A Optimizing the latent space dimension toward evaluation metrics for the different models

In our work, the latent dimensions are fixed between the different models in order to preserve comparability (different latent spaces correspond to a different number of trainable parameters). For the 2D shape models, a latent dimension of 512 was chosen, since it is the smallest model corresponding to specific thresholds: specificity $\le 2.6$mm and generalization ability $\le 1.5$mm for all models when the largest possible training set is used. For the 3D models, 1024 was chosen similarly setting both cutoff values at 0.5mm. However, choosing the latent dimensionality for each model separately is not trivial, since a trade-off between specificity and generalization, as well as computational feasibility needs to be obtained. A good value might result in a good result for specificity for one model, but lead to bad generalization ability in that same model or be less suitable for other models. Here, we investigated that property exemplary for the use-case of the 2D shapes. The tested dimensions are 128, 256, 512, 1024, 1500. The experimental setup is corresponding to the previous experiments using the same training set sizes and a multi-fold validation. The results for the three highest dimensions are presented in Fig. 7 since 512 is the first size delivering optimal results for at least one of the models. Interestingly, larger dimensions are not always contributing to better generalization ability: the GAN and AE models yield best results using latent vectors of size 512, contrary to the VAE and DAE approaches, that achieve slightly better results with a latent dimension of size 1500. Another important observation is that specificity is not best, when generalization is best. However, overall those different dimensions deliver fairly similar results, such that 512 remains a good choice for the means of comparability and computational efficacy.

## B Additional metrics for the evaluation of appearance models

Figure 8 presents additional metrics for the evaluation of the appearance modeling approaches: Mean squared error (MSE) and structured similarity index (SSIM). Results comparable to the L1 metric from Fig. 5.

## Footnotes

1 brain-development.org/ixi-dataset/.

## References

[1] Bône A, Louis M, Colliot O, Durrleman S (2019) Learning Low-Dimensional Representations of Shape Data Sets with Diffeomorphic Autoencoders. In: information processing in medical imaging, pp. 195–207

[2] Chen M, Shi X, Zhang Y, Wu D, Guizani M (2017) Deep Features Learning for Medical Image Analysis with Convolutional Autoencoder Neural Network. IEEE Trans Big Data. pp. 1–1

[3] Cootes TF, Edwards GJ, Taylor CJ (1998) Active appearance models. In: European conference on computer vision, pp. 484–498. Springer

[4] Cootes TF, Taylor CJ, Cooper DH, Graham J (1995). Active shape models-their training and application. Comput Vis Image Underst.

[5] Davatzikos C, Tao X, Shen D (2003) Hierarchical active shape models, using the wavelet transform. IEEE Trans Med Imaging p 10.1109/TMI.2003.809688 12760558

[6] Elbattah M, Loughnane C, Guérin JL, Carette R, Cilia F, Dequen G (2021). Variational autoencoder for image-based augmentation of eye-tracking data. J Imag.

[7] Frid-Adar M, Diamant I, Klang E, Amitai M, Goldberger J, Greenspan H (2018). GAN-based synthetic medical image augmentation for increased CNN performance in liver lesion classification. Neurocomputing.

[8] Ghosh P, Sajjadi MSM, Vergari A, Black M, Scholkopf B (2020) From variational to deterministic autoencoders. In: international conference on learning representations. https://openreview.net/forum?id=S1g7tpEYDS

[9] Goodfellow I, Pouget-Abadie J, Mirza M, Xu B, Warde-Farley D, Ozair S, Courville A, Bengio Y (2014) Generative Adversarial Nets. In: advances in neural information processing systems. 27, pp 2672–2680

[10] Guan S, Loew M (2020) An Internal Cluster Validity Index Using a Distance-based Separability Measure. In: 2020 IEEE 32nd international conference on tools with artificial intelligence (ICTAI), pp 827–834

[11] Hammers A, Allom R, Koepp MJ, Free SL, Myers R, Lemieux L, Mitchell TN, Brooks DJ, Duncan JS (2003). Three-dimensional maximum probability atlas of the human brain, with particular reference to the temporal lobe. Hum Brain Mapp.

[12] Heimann T, Meinzer HP (2009). Statistical shape models for 3d medical image segmentation: a review. Med Image Anal.

[13] Hu Y, Gibson E, Ahmed HU, Moore CM, Emberton M, Barratt DC (2015). Population-based prediction of subject-specific prostate deformation for MR-to-ultrasound image registration. Med Image Anal.

[14] Hufnagel H, Pennec X, Ehrhardt J, Ayache N, Handels H (2008). Generation of a statistical shape model with probabilistic point correspondences and the expectation maximization-iterative closest point algorithm. Int J Comput Assist Radiol Surg.

[15] Karimi D, Samei G, Kesch C, Nir G, Salcudean SE (2018). Prostate segmentation in mri using a convolutional neural network architecture and training strategy based on statistical shape models. Int J Comput Assist Radiol Surg.

[16] Kingma D, Welling M (2014) Auto-Encoding Variational Bayes. In: international conference on learning representations

[17] Kirschner M, Becker M, Wesarg S (2011) 3D Active Shape Model Segmentation with Nonlinear Shape Priors. In: medical image computing and computer-assisted intervention – MICCAI 2011, pp. 492–499 10.1007/978-3-642-23629-7_60 21995065

[18] Krüger J, Ehrhardt J, Handels H (2017). Statistical appearance models based on probabilistic correspondences. Med Image Anal.

[19] Larsen ABL, Sønderby SK, Larochelle H, Winther O (2016) Autoencoding beyond pixels using a learned similarity metric. In: international conference on machine learning, pp 1558–1566

[20] Milletari F, Rothberg A, Jia J, Sofka M, Descoteaux M, Maier-Hein L, Franz A, Jannin P, Collins DL, Duchesne S (2017). Integrating statistical prior knowledge into convolutional neural networks. Medical Image Computing and Computer Assisted Intervention - MICCAI 2017, LNCS.

[21] Oord AV, Kalchbrenner N, Kavukcuoglu K (2016) Pixel Recurrent Neural Networks. In: proceedings of The 33rd international conference on machine learning, pp 1747–1756. PMLR

[22] Rezende D, Mohamed S (2015) Variational Inference with Normalizing Flows. In: proceedings of the 32nd international conference on machine learning, pp 1530–1538. PMLR

[23] Royston JP (1982). An extension of shapiro and Wilk’s W test for normality to large samples. J R Stat Soc Ser C Appl Stat..

[24] Schlegl T, Seeböck P, Waldstein SM, Langs G, Schmidt-Erfurth U (2019). F-AnoGAN: fast unsupervised anomaly detection with generative adversarial networks. Med Image Anal.

[25] Sudre CH, Li W, Vercauteren T, Ourselin S, Jorge Cardoso M (2017) Generalised Dice Overlap as a Deep Learning Loss Function for Highly Unbalanced Segmentations. In: deep learning in medical image analysis and multimodal learning for clinical decision support, pp 240–248 10.1007/978-3-319-67558-9_28 PMC7610921 34104926

[26] Uzunova H, Handels H, Ehrhardt J (2021) Guided Filter Regularization for Improved Disentanglement of Shape and Appearance in Diffeomorphic Autoencoders. In: medical imaging with deep learning – MIDL

[27] Uzunova H, Kruse J, Kaftan P, Wilms M, Forkert ND, Handels H, Ehrhardt J (2021) Analysis of Generative Shape Modeling Approaches: Latent Space Properties and Interpretability. In: Bildverarbeitung Für Die Medizin 2021: proceedings, German workshop on medical image computing, Regensburg, march 7-9, 2021, pp. 344–349

[28] Uzunova H, Schultz S, Handels H, Ehrhardt J (2019). Unsupervised pathology detection in medical images using conditional variational autoencoders. Int J Comput Assist Radiol Surg.

[29] Uzunova H, Wilms M, Handels H, Ehrhardt J (2017) Training CNNs for Image Registration from Few Samples with Model-based Data Augmentation. In: medical image computing and computer assisted intervention - MICCAI 2017, pp. 223–231

[30] van Ginneken B, Stegmann MB, Loog M (2006). Segmentation of anatomical structures in chest radiographs using supervised methods: a comparative study on a public database. Med Image Anal.

[31] Wilms M, Ehrhardt J, Forkert ND (2020) A Kernelized Multi-level Localization Method for Flexible Shape Modeling with Few Training Data. In: medical image computing and computer assisted intervention – MICCAI 2020, pp. 765–775

[32] Wilms M, Handels H, Ehrhardt J (2017). Multi-resolution multi-object statistical shape models based on the locality assumption. Med Image Anal.

[33] Yu X, Zhou F, Chandraker M (2016) Deep deformation network for object landmark localization. In: B. Leibe, J. Matas, N. Sebe, M. Welling (eds.) European conference on computer vision – ECCV 2016, LNCS, pp. 52–70. Springer

